# Potential Dysphagia Following COVID-19 Infection Due to Cervical Osteophytes: A Case Report

**DOI:** 10.7759/cureus.94248

**Published:** 2025-10-09

**Authors:** Shuji Matsumoto, Rintaro Ohama, Takashi Hoei, Ryuji Tojo, Toshihiro Nakamura

**Affiliations:** 1 Center for Medical Science, Ibaraki Prefectural University of Health Sciences, Ami, JPN; 2 Department of Rehabilitation and Physical Medicine, Kagoshima University Graduate School of Medical and Dental Sciences, Kagoshima, JPN; 3 Department of Rehabilitation, Kagoshima University Hospital, Kagoshima, JPN; 4 Department of Rehabilitation, Acras Central Hospital, Kagoshima, JPN

**Keywords:** compensatory swallowing technique, covid-19, dysphagia, osteophytes, postural adjustment

## Abstract

The epiglottis separates the pharynx from the larynx by flipping upward to prevent swallowed material from entering the airway. Cervical vertebral osteophytes can interfere with this movement, potentially leading to dysphagia. Videofluoroscopic (VF) and videoendoscopic (VE) examinations of swallowing are valuable tools for understanding the pathophysiology of dysphagia and for formulating appropriate rehabilitation plans.

A 77-year-old man with pre-existing dysphagia for solid foods was hospitalized for coronavirus disease 2019 (COVID-19). He stabilized with antiviral and supportive therapy but developed aspiration pneumonia on the sixth day of hospitalization. Rehabilitation therapy was initiated on day 7.

Upon admission, the patient was kept fasting, and a VF swallowing study was performed. This revealed cervical vertebral osteophytes at the C3/4 and C4/5 levels that impeded epiglottic inversion and hindered bolus passage through the pyriform sinus. It was considered that dysphagia became apparent following COVID-19 infection, leading to aspiration pneumonia. Surgery was not performed, and progressive feeding training was initiated. Under VE evaluation, compensatory swallowing strategies were explored. Pharyngeal residue was reduced by adopting a forward-leaning posture and performing chin-down maneuvers. Alternating swallows of unthickened liquids further minimized pharyngeal residue, which the patient could expel independently. By combining the forward-leaning and chin-down postures, alternating swallows of unthickened liquids, and self-expulsion after swallowing, the patient successfully resumed oral intake of three regular meals and was discharged home.

The primary cause of dysphagia in this case was presumed to be cervical vertebral osteophytes, with symptoms becoming apparent following COVID-19 infection. It is important to recognize that dysphagia can arise from cervical spine deformities even in the absence of neurological abnormalities. In this patient, compensatory swallowing techniques such as the forward-leaning posture and chin-down position promoted favorable deformation of the pharyngeal cavity and reduced pharyngeal residue. This case underscores the importance of identifying the underlying cause of dysphagia and implementing appropriate risk mitigation strategies.

## Introduction

The epiglottis is composed of elastic cartilage covered by mucous membrane and is attached to the entrance of the larynx. It forms the upper margin of the larynx and projects vertically behind the tongue and hyoid bone. During swallowing, the epiglottis flips upward to separate the pharynx from the larynx, thereby preventing bolus entry into the airway [1]. Impaired epiglottic elevation increases pharyngeal residue and the risk of laryngeal penetration or aspiration. One cause of such impairment is posterior pharyngeal wall protrusion resulting from osteophytes formed on the cervical vertebral bodies [2].

To assess epiglottic inversion inhibition due to posterior wall protrusion, direct visualization using videoendoscopic (VE) examination of swallowing (VE) is valuable [3]. Additionally, videofluoroscopic (VF) examination of swallowing under X-ray fluoroscopy enables evaluation of aspiration risk by observing epiglottic motion during swallowing [4]. Surgical removal of cervical vertebral osteophytes has been reported to improve dysphagia in some patients [5]; however, other cases have shown improvement through conservative management with posture adjustments [6]. This report describes a case in which cervical vertebral osteophytes caused poor epiglottic inversion and obstruction of bolus passage through the pyriform sinus in a patient who developed aspiration pneumonia during treatment for coronavirus disease 2019 (COVID-19). Dysphagia was successfully improved through compensatory swallowing techniques (conservative therapy) guided by VF evaluation.

## Case presentation

**Figure 1:**
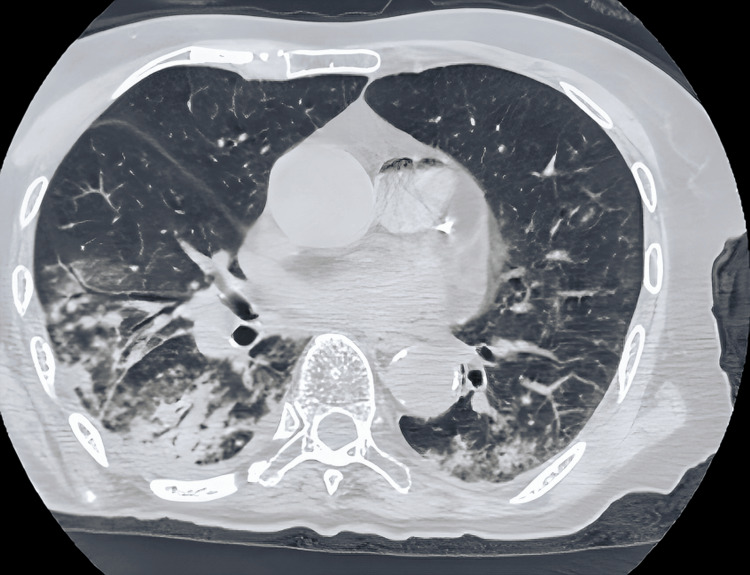
Chest CT at the onset of aspiration pneumonia (sixth day of hospitalization) Consolidation (arrow) and bronchial wall thickening are observed in the basal segments of both lungs. CT: computed tomography.

A 77-year-old man was admitted with a diagnosis of coronavirus disease 2019 (COVID-19). His past medical history included hypertension. Regarding smoking history, the patient was a former smoker, having smoked approximately 1-5 cigarettes per day until the age of 60. He was completely independent in all activities of daily living. His main complaints upon admission were, “I want to eat regular rice” and “I want to go home.” He presented with fever and dyspnea, and pulse oximetry revealed hypoxemia (SpO₂ 92%). COVID-19 was confirmed by polymerase chain reaction (PCR) testing, and the patient was subsequently hospitalized. Chest computed tomography (CT) showed no obvious signs of pneumonia. Treatment included remdesivir (200 mg on day 1, followed by 100 mg once daily intravenously on days 2-5) and oxygen therapy. Endotracheal intubation was not required. The fever resolved on the second day of hospitalization, and the patient remained stable thereafter. However, aspiration pneumonia developed on the sixth day of hospitalization (Figure 1).

Subsequently, aspiration pneumonia recurred during hospitalization, requiring fasting and management with continuous intravenous infusion. Temporary oxygen therapy at 5 L/min was administered, after which the patient’s respiratory status gradually stabilized, and oxygen supplementation was discontinued on the 18th day of hospitalization. Rehabilitation therapy began on the seventh day and was conducted daily for approximately one hour, provided the patient’s body temperature remained below 37.5 °C (99.5 °F). Neurological and neuropsychological evaluations revealed no abnormalities, including no evidence of paralysis, sensory disturbance, or cranial nerve deficits. Prior to publication, the study’s ethical considerations were fully explained to the patient, and written consent for publication was obtained.

Hospitalization course

Until the 18th day of hospitalization, the patient remained bedridden for most of the day, with opportunities to get out of bed limited to rehabilitation sessions. In practice, he was able to walk independently under supervision within the hospital and could independently manage bed mobility, sitting balance, and transfers. Oxygen saturation during exertion remained between 90% and 94% on room air. The patient was cooperative with rehabilitation but showed easy fatigability. Occasional wet coughs were observed during conversation, though he was able to clear secretions through self-expectoration.

Speech-language pathology (ST) rehabilitation interventions began on the seventh day of hospitalization, following the initial episode of aspiration pneumonia. The patient continued to consume three jelly-form meals orally; however, on the 11th day, his respiratory status deteriorated again, necessitating fasting and transition to peripheral intravenous nutrition. Oral intake was gradually resumed on the 19th day, starting with one jelly per day under ST supervision. Before hospitalization, the patient had reported difficulty swallowing solid or hard foods, often spitting them out or eating only after cutting them into very small pieces.

**Figure 2:**
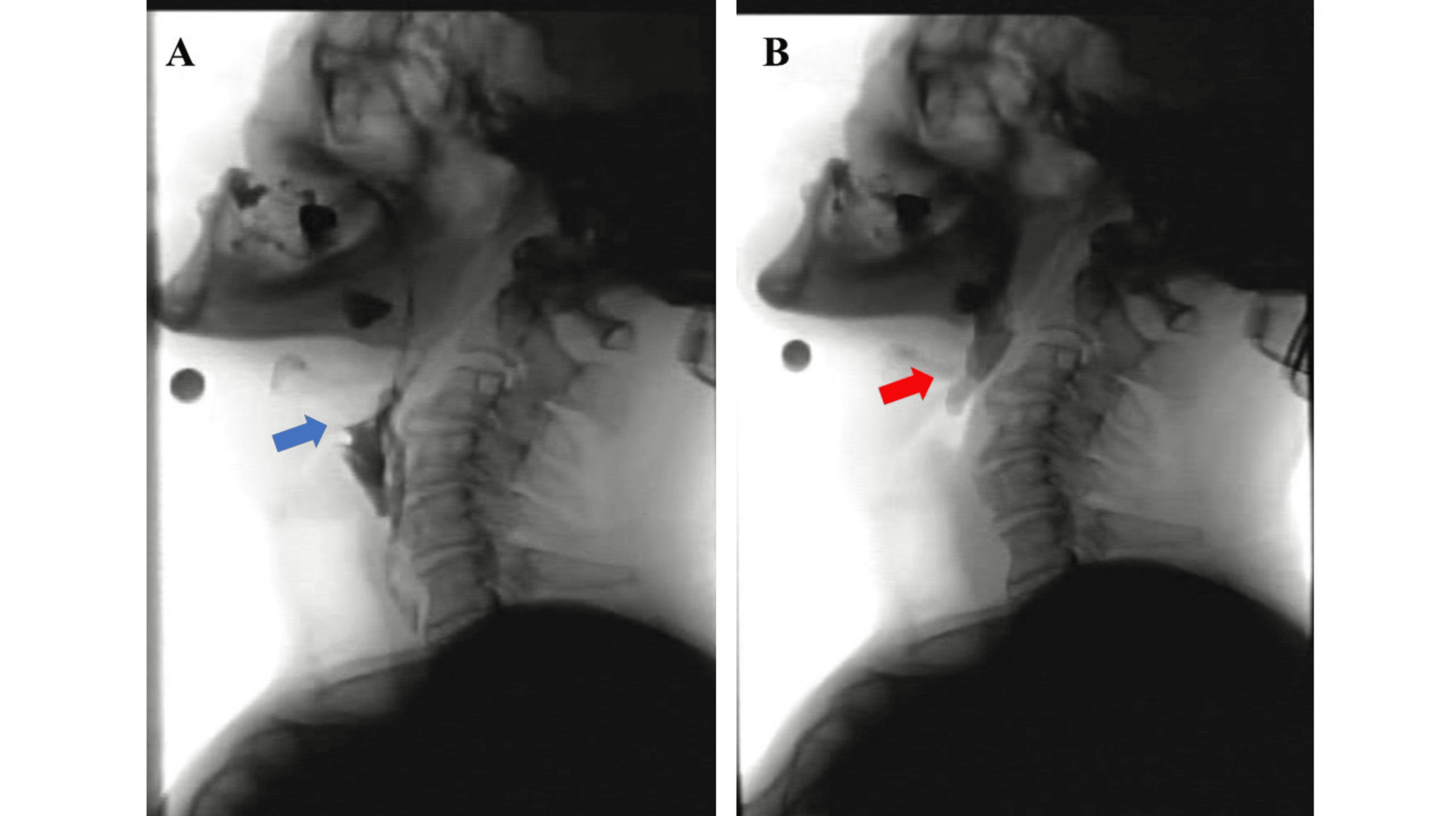
Videofluoroscopic (VF) examination of swallowing (A) Laryngeal closure insufficiency. (B) Food residue in the epiglottis vallecula. No obvious laryngeal invasion or aspiration is noted during the swallowing reflex; however, the epiglottis did not retract (blue arrow), and residual material remained in the pharynx after swallowing (red arrow).

**Figure 3:**
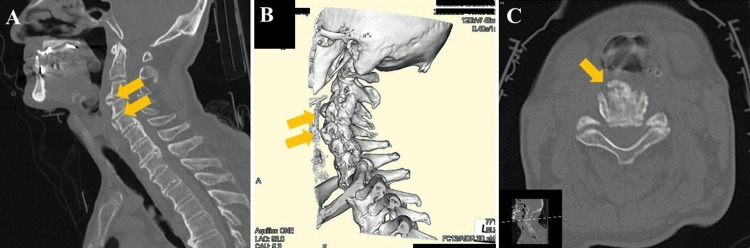
CT imaging (A) Sagittal CT image. (B) 3D CT image. (C) Axial CT image. Evidence of ossification of the anterior longitudinal ligament at the C3/4 and C4/5 is observed (yellow arrow). CT: computed tomography.

On the 24th day of hospitalization, VF was performed. No abnormalities were observed during the antecedent, preparatory, or oral phases. However, osteophytes were identified on the anterior surface of the cervical vertebral bodies at the C3-C5 levels. These osteophytes interfered with epiglottic movement during swallowing, resulting in incomplete laryngeal closure (Figure 2A). The anterior-superior movement of the hyoid bone during swallowing was also reduced, leading to incomplete opening and obstruction near the esophageal entrance. No esophageal reflux was observed. Although no definite laryngeal penetration or aspiration was seen with any food consistency, including thin-thickened liquids, yogurt, or jelly, as classified by the Japanese Society of Dysphagia Rehabilitation [7], moderate pharyngeal residue was noted in the epiglottic vallecula (Figure 2B). To mitigate aspiration risk from pharyngeal residue, a compensatory swallowing technique using a chin-down posture was attempted [8], resulting in a slight reduction in residue. A subsequent cervical CT scan revealed ossification of the anterior longitudinal ligament at the C3/4 and C4/5 levels (Figure 3). The patient was offered surgical removal of the osteophytes but declined, opting instead for conservative management through stepwise dietary training.

Starting on day 26, the patient began oral intake of pureed food during lunch under the supervision of a speech therapist. No signs of aspiration were observed. By day 28, the patient progressed to self-feeding with therapist supervision. As compensatory swallowing techniques, the patient maintained a seated position supported by the ischial tuberosity, leaned the upper torso forward during swallowing (forward-leaning posture), and used the chin-down technique [5]. Clearing the throat after swallowing to remove residual material was incorporated into self-feeding, and habit formation was encouraged. No aspiration was noted, and by day 30, the patient advanced to three oral meals per day.

On day 32, VE evaluation was performed. Residual food was mainly seen in the epiglottic vallecula when swallowing paste food while seated. Alternating swallows with liquids reduced pharyngeal residue, with unthickened liquids further minimizing it. No aspiration or laryngeal penetration was observed. Comparison of pharyngeal residue before and after adopting the forward-leaning and chin-down posture showed a clear reduction in residual material (Figure 3). From day 32 onward, the patient was instructed to consistently use these postures and alternate swallows with liquids. Thickened liquids were discontinued. No aspiration was noted thereafter. On day 37, the diet was advanced to soft-cooked rice and bite-sized solid foods. Although chewing took longer, swallowing remained stable. By day 41, the patient resumed regular rice and solid food, returning to the same diet as before hospitalization. The patient reported that leaning forward and performing the chin-down maneuver made swallowing easier than before the illness. There were no episodes of regurgitation or avoidance of solid food, and the patient maintained stable oral intake. On day 43, the goal of safely consuming regular meals was achieved, and the patient was discharged.

**Figure 4:**
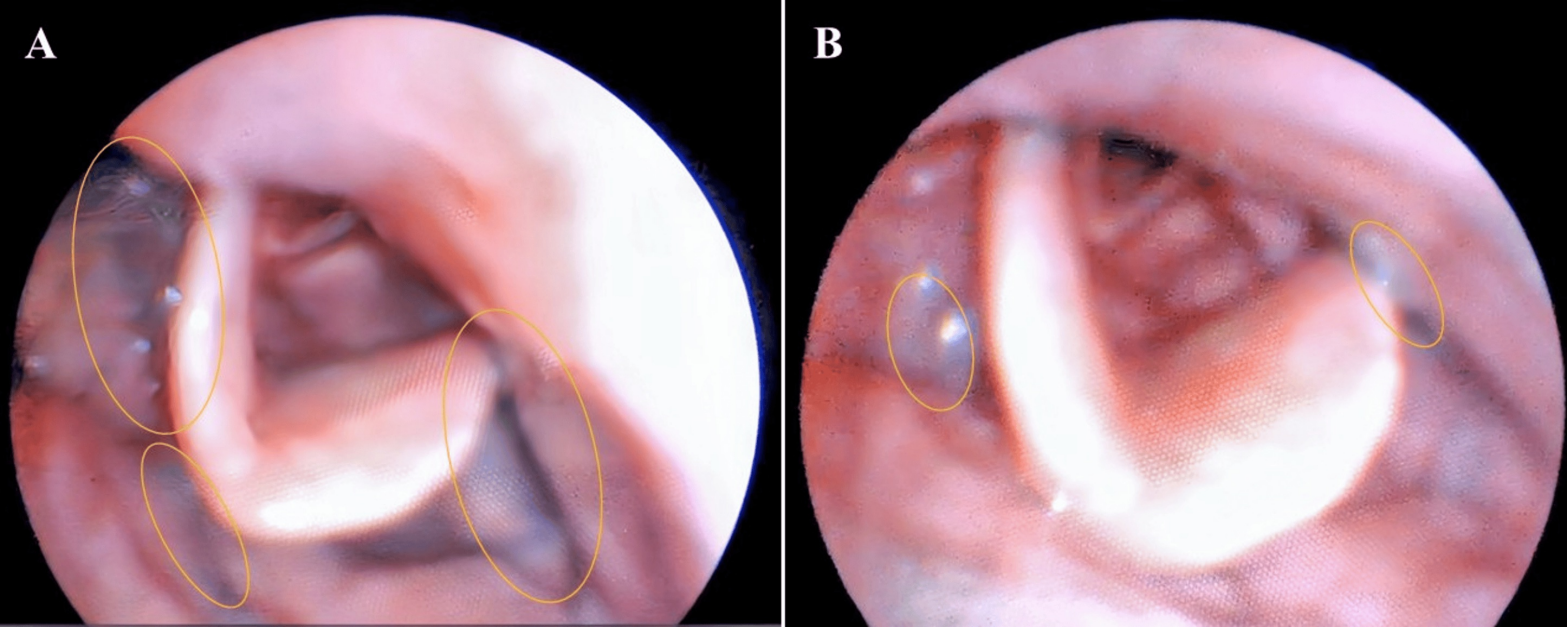
Videoendoscopic (VE) examination of swallowing (A) Without postural adjustments. (B) With postural adjustments. Pharyngeal residues (yellow circles) after swallowing were reduced in the epiglottic valley when adjusting to a forward-head posture and performing chin-down, compared to without postural adjustment.

## Discussion

Ossification of the anterior longitudinal ligament can present with progressive dysphagia as the primary symptom [9]. Although this patient had no history of aspiration pneumonia prior to hospitalization, the pre-existing difficulty in swallowing solid foods suggested an underlying dysphagia. COVID-19 infection has been reported to potentially exacerbate dysphagia [10,11]. In this case, aspiration pneumonia likely developed early during hospitalization due to transient respiratory deterioration during treatment, combined with reduced immunity and an impaired cough reflex from prolonged bed rest [12]. While tracheal intubation during COVID-19 treatment is a known cause of dysphagia [13,14], it was not performed in this case, and no neurological disorders associated with dysphagia were identified. VF examination confirmed that cervical vertebral osteophytes impeded epiglottic inversion, resulting in impaired bolus transport characteristic of pharyngeal-phase dysphagia.

Several factors may explain why COVID-19 infection caused previously latent dysphagia to manifest. First, “disuse” can be considered [15]. Although less likely, given the relatively short recovery period of COVID-19, disuse resulting from recurrent aspiration pneumonia and subsequent physical deconditioning may have contributed. Second, COVID-19 may exert direct neurotropic or neuroinvasive effects [15]. Viral invasion of the central nervous system could impair cortical adaptation following infection, leading to reduced pharyngeal swallowing function. Even in patients who were not intubated, reports have described COVID-19-related swallowing disorders due to these neuroinvasive properties [16]. Therefore, the effect of COVID-19 on overall physical and neurological function should not be underestimated. Third, alterations in pulmonary function due to COVID-19 may also play a role [16]. COVID-19-induced lung injury could have exacerbated pre-existing respiratory impairment, further worsening swallowing coordination. In contrast, there was no evidence that COVID-19 caused cranial neuropathy or myositis in this patient. No symptoms or signs suggested involvement of the facial, glossopharyngeal, or vagus nerves, nor were anosmia, ageusia, muscle pain, or elevated serum creatine kinase levels observed.

The protrusion of the posterior pharyngeal wall caused by cervical osteophytes is an organic disorder, and surgical removal of the osteophytes is generally expected to improve dysphagia [5,17]. However, given the patient’s advanced age and his preference against surgery, conservative management was selected. Ikegami et al. reported that using VF to assess and adjust posture affecting epiglottic movement during swallowing, specifically self-feeding in a seated position with sacral support and neck flexion, improved pharyngeal clearance by expanding the pharyngeal cavity [6]. In the present case, VF confirmed that the bone spur obstructed epiglottic movement, preventing complete inversion. Adopting a forward-leaning posture during meals modified the structural angle of the pharyngolaryngeal region, moderated bolus flow velocity from the nasopharynx to the hypopharynx, and enhanced compensatory pharyngolaryngeal function [18]. Additionally, the chin-down position deformed the pharyngeal cavity during swallowing, reduced inhibition of epiglottic inversion, and compensated for limited posterior tongue-base movement [8].

To facilitate the transition to solid food intake, further evaluation using VE, which provides a detailed visualization of organic morphological abnormalities of the pharynx and larynx, was considered necessary [19]. VE confirmed that adopting a forward-leaning posture and chin-down position reduced pharyngeal residue. Adhesion to the mucosa was less pronounced with non-thickened liquids than with thickened liquids, highlighting the effectiveness of alternating swallows with liquids. In addition to the lateral view assessment provided by VF, the complementary use of VE, which offers three-dimensional information, proved valuable in evaluating compensatory swallowing techniques for solid food intake [20]. This comprehensive approach ultimately enabled the patient to resume solid food consumption and return home.

The dysphagia in this case was presumed to have been present before hospitalization, with symptoms due to cervical osteophytes becoming apparent following COVID-19 infection. However, VF enabled objective assessment of swallowing function, and VE allowed the identification of effective compensatory swallowing techniques. This case highlights the importance of a comprehensive evaluation of swallowing function, including gathering information about the patient’s pre-hospitalization dietary habits, considering potential causes of dysphagia during assessment, and exploring appropriate risk mitigation strategies.

## Conclusions

In this case of aspiration pneumonia following COVID-19 infection, a VF study revealed impaired epiglottic inversion and obstruction of bolus passage through the pyriform sinus caused by cervical vertebral osteophytes. The dysphagia was presumed to have existed before hospitalization but became apparent after COVID-19 infection, leading to aspiration pneumonia. It is important to recognize that dysphagia can manifest following COVID-19 infection and may also result from cervical spine deformities, even in the absence of neurological abnormalities. This case suggests that combining VF and VE assessments with posture adjustments and compensatory swallowing techniques can enhance epiglottic movement and reduce the risk of aspiration and choking. The complementary use of VF and VE supports the potential for conservative management to improve dysphagia caused by structural abnormalities.
